# ^18^F-FDG PET Imaging of Murine Atherosclerosis: Association with Gene Expression of Key Molecular Markers

**DOI:** 10.1371/journal.pone.0050908

**Published:** 2012-11-30

**Authors:** Anne Mette Fisker Hag, Sune Folke Pedersen, Christina Christoffersen, Tina Binderup, Mette Munk Jensen, Jesper Tranekjær Jørgensen, Dorthe Skovgaard, Rasmus Sejersten Ripa, Andreas Kjaer

**Affiliations:** 1 Cluster for Molecular Imaging, Faculty of Health and Medical Sciences and Department of Clinical Physiology, Nuclear Medicine & PET, Rigshospitalet, University of Copenhagen, Copenhagen, Denmark; 2 Department of Clinical Biochemistry, Rigshospitalet, Copenhagen, Denmark; University of Freiburg, Germany

## Abstract

**Aim:**

To study whether ^18^F-FDG can be used for *in vivo* imaging of atherogenesis by examining the correlation between ^18^F-FDG uptake and gene expression of key molecular markers of atherosclerosis in apoE^−/−^ mice.

**Methods:**

Nine groups of apoE^−/−^ mice were given normal chow or high-fat diet. At different time-points, ^18^F-FDG PET/contrast-enhanced CT scans were performed on dedicated animal scanners. After scans, animals were euthanized, aortas removed, gamma counted, RNA extracted from the tissue, and gene expression of chemo (C-X-C motif) ligand 1 (CXCL-1), monocyte chemoattractant protein (MCP)-1, vascular cell adhesion molecule (VCAM)-1, cluster of differentiation molecule (CD)-68, osteopontin (OPN), lectin-like oxidized LDL-receptor (LOX)-1, hypoxia-inducible factor (HIF)-1α, HIF-2α, vascular endothelial growth factor A (VEGF), and tissue factor (TF) was measured by means of qPCR.

**Results:**

The uptake of ^18^F-FDG increased over time in the groups of mice receiving high-fat diet measured by PET and *ex vivo* gamma counting. The gene expression of all examined markers of atherosclerosis correlated significantly with ^18^F-FDG uptake. The strongest correlation was seen with TF and CD68 (p<0.001). A multivariate analysis showed CD68, OPN, TF, and VCAM-1 to be the most important contributors to the uptake of ^18^F-FDG. Together they could explain 60% of the ^18^F-FDG uptake.

**Conclusion:**

We have demonstrated that ^18^F-FDG can be used to follow the progression of atherosclerosis in apoE^−/−^ mice. The gene expression of ten molecular markers representing different molecular processes important for atherosclerosis was shown to correlate with the uptake of ^18^F-FDG. Especially, the gene expressions of CD68, OPN, TF, and VCAM-1 were strong predictors for the uptake.

## Introduction

The use of positron emission tomography (PET) for visualization of atherosclerosis has been evolving over the last decade. The visualization of the vulnerable plaque using the tracer ^18^F-FDG is promising [1]–[4], and also ^18^F-FDG uptake as a surrogate marker for atherosclerotic disease activity shows potential [5], [6]. The pre-clinical *in vivo* research of ^18^F-FDG has mainly focused on rabbits [2], [7], [8]. As transgenic mouse models have shown their value in atherosclerosis research we have focused on developing the technique of small animal PET for *in vivo* imaging of atherosclerosis in mice. A study published in 2011 [9] used ^18^F-FDG for *in vivo* imaging of mice and their results suggest that the method can be used to follow the development of atherosclerosis in murine models.

Atherogenesis is a complex disease characterized by inflammation [10], [11] and many molecular processes are involved. In this article, we focus on five of these processes represented by different molecular markers: (a) monocyte and macrophage recruitment represented by chemo (C-X-C motif) ligand 1 (CXCL-1), monocyte chemoattractant protein (MCP)-1, and vascular cell adhesion molecule (VCAM)-1, (b) macrophages and inflammation represented by cluster of differentiation molecule (CD)-68 and osteopontin (OPN), (c) scavenger receptors represented by lectin-like oxidized LDL-receptor (LOX)-1, (d) hypoxia represented by hypoxia-inducible factor (HIF)-1α, HIF-2α and vascular endothelial growth factor A (VEGF), and (e) thrombogenicity represented by tissue factor (TF).

The aim of the study was to evaluate the uptake of ^18^F-FDG in the aorta of apolipoprotein E knockout (apoE^−/−^) mice and to correlate the tracer uptake with gene expression of the molecular markers mentioned above in order to test the hypothesis that ^18^F-FDG can be used for *in vivo* imaging of key atherosclerotic processes.

## Materials and Methods

### Ethical Statement

All care and all experimental procedures were performed under the approval of the Animal Experiments Inspectorate in Denmark (permit number 2011/561–14). All efforts were made to minimize suffering.

### Experimental Model

Homozygous apoE^−/−^ mice (B6.129P2-*Apoe* ^tm1Unc^N11) were purchased from Taconic (Taconic Europe, Denmark). The mice were 8 weeks old upon initiation of the experiment.

The mice were housed under controlled humidity, temperature, and light cycle conditions, and had free access to food and water throughout the course of experiments.

The mice were divided into nine groups. The characteristics of the groups are shown in Table 1. All animals were scanned once and then sacrificed. One group was scanned and sacrificed at the beginning of the experiment as a baseline group (0 weeks). Four other groups received normal chow for 8, 16, 24 or 32 weeks (8 weeks, 16 weeks, 24 weeks or 32 weeks) before scanning and sacrifice. The last four groups received a high-fat Western type diet for 8, 16, 24 or 32 weeks (8 weeks+diet, 16 weeks+diet, 24 weeks+diet or 32 weeks+diet). The high-fat Western type diet contained 21% fat and 0.21% cholesterol (diet #TD12079B, Research Diets, Inc., USA).

**Table 1:** Basic characteristics of investigated animals.

Group	No. of animals^1^	Age	Weeks on high-fat diet	Weight±SD (g)	Cholesterol±SD (nM)
0 weeks	8 (11/11)	8	–	22.9±2.0	13.4±4.1
8 weeks	12 (12/12)	16	–	31.6±1.2***	13.9±1.9
16 weeks	11 (12/12)	24	–	33.7±0.9***	21.0±4.9*
24 weeks	11 (11/11)	32	–	35.6±2.2***	18.6±2.4*
32 weeks	12 (12/12)	40	–	35.7±2.1***	15.7±2.3
8 weeks+diet	12 (12/12)	16	8	34.0±2.1***	21.9±5.0**, ###
16 weeks+diet	11 (12/12)	24	16	42.7±5.2***, ##	31.0±5.1***, ##
24 weeks+diet	11 (11/12)	32	24	47.8±4.5***, ###	32.4±5.3***, ###
32 weeks+diet	11 (11/11)	40	32	47.6±5.7***, ###	33.4±4.8***, ###

1 Number of animal imaged and weighed (number of animal tested for cholesterol/number of animals tested for gene expression).

* *p*<0.05.

** *p*<0.01.

*** *p*<0.001 vs. 0 weeks

## *p*<0.01.

### *p*<0.001 vs. diet (all *p*-values were Bonferroni corrected).

### Experimental Protocol

The animals were fasted overnight prior to the scan [12]. Before injection, 30 minutes after injection and during the scans, the animals were anaesthetized using a mixture of 3% sevoflurane (Abbott Scandinavia AB, Sweden) mixed with 35% O_2_ in N_2_ by breathing through a nose cone. The mice were kept at a temperature of approximately 32°C from the time of the injection to the scans were executed.

^18^F-FDG was obtained from our own production facilities (Rigshospitalet, Denmark). The exact concentration of the ^18^F-FDG solution was measured in a Radioisotope Calibrator ARC-120 (Amersham, United Kingdom). 20.1±4.8 MBq in 0.3 mL physiological saline was administered i.v. (slow injection over several minutes) to the mice in a lateral vein using a vein catheter (BD Vasculon™Plus, Becton Dickinson A/S, Denmark). Immediately after this, 0.2–0.3 mL of a long circulating emulsion formulation containing an iodinated triglyceride (Fenestra VC®, ART Advanced Research Technologies Inc., Canada) was administered through the same vein catheter. The mice remained anaesthetized for approximately 30 minutes after the injection to limit the up-take of ^18^F-FDG in brown fat [12].

Three hours after injection, the animals were placed in a prone position on the acquisition bed and a 30 minutes PET scan was acquired, followed by a CT scan. The same acquisition bed was used for both scans, so the animals remained in precisely the same position during both scans. The animals were then sacrificed by decapitation. The blood was collected and centrifuged (3,200 RPM for 10 minutes) and plasma was transferred to a fresh tube and store at −20°C. The aorta was removed with care taken not to include any surrounding tissue and placed in RNAlater® (Ambion Europe Limited, United Kingdom). Subsequently, the aorta was gamma counted and stored at 4°C. The following day, RNAlater® was removed and the samples stored at −80°C until RNA extraction.

### CT Protocol

CT data were acquired with a MicroCAT II tomography (Siemens Medical Solutions, USA). The X-ray tube with a 0.5 mm aluminium filter was set at 60 kVp, a tube current of 500 µA, and an exposure time of 310 ms per projection. A set of 360 projections was used for a full 360° scan. Images were reconstructed using the COBRA real-time reconstruction with the Sheep-Logan filter. The voxel size was 0.095×0.095×0.095 mm^3^.

### PET Protocol

PET data were acquired with a microPET Focus 120 scanner (Siemens Medical Solutions). The energy window of the emission scan was set to 350–650 keV with a time resolution of 6 ns. The acquired emission dataset was automatically stored in listmode. All listmode data were post-processed into 128×144×95 sinograms using a span of three and a ring difference of 47. The sinograms were then reconstructed using 3D Maximum *a posteriori* (MAP) algorithms [13]. The voxel size was 0.3×0.3×0.3 mm^3^ and in the centre of field of view, the resolution was 1.2 mm full-width at half-maximum. The emission data were normalized and decay and dead time corrected. The system was calibrated to provide the quantification unity in becqerel per millilitre.

**Figure 1:**
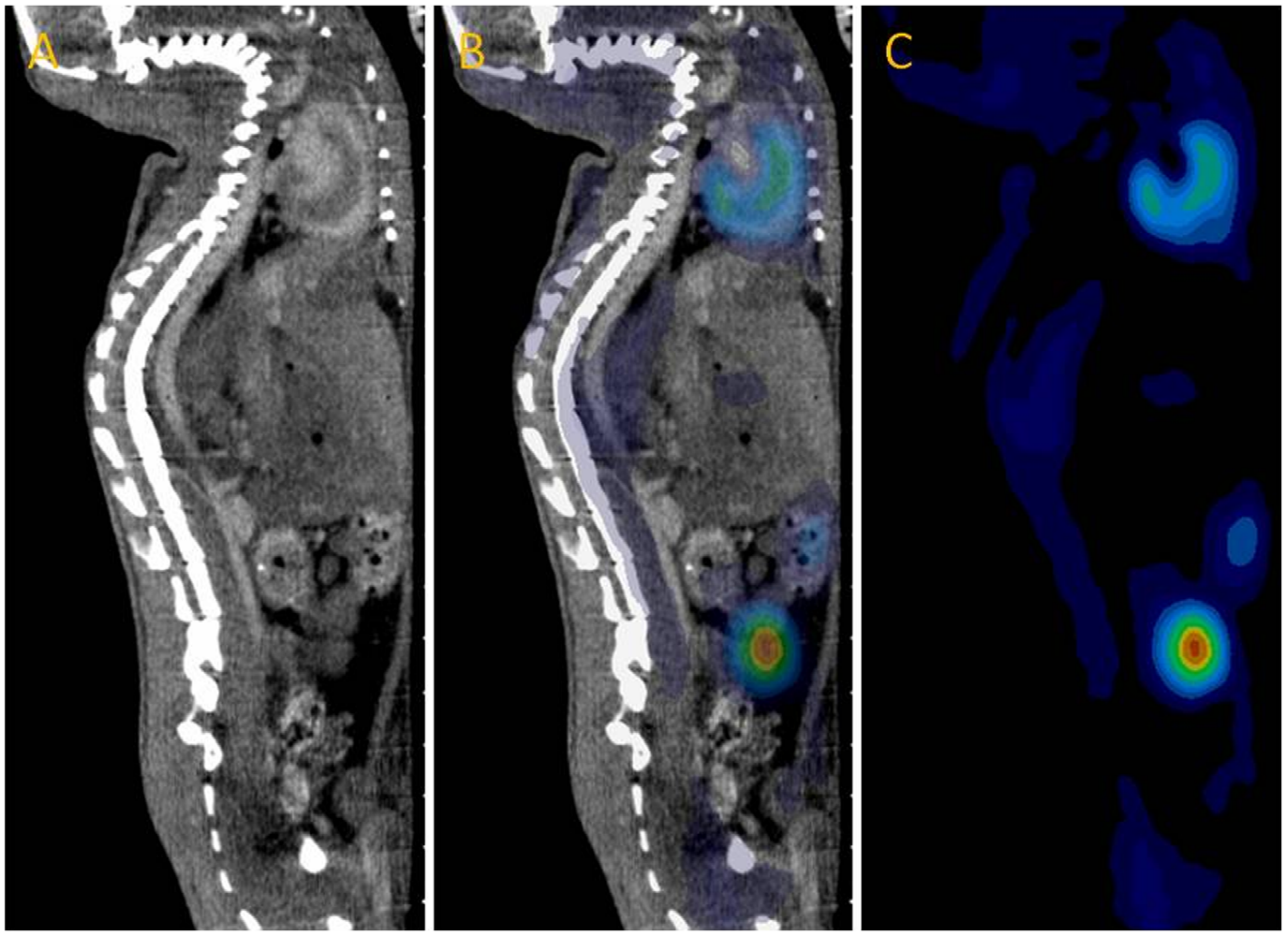
CT, fused PET/CT, and PET images. **A** Contrast-enhanced CT image. **B** Fused PET/CT image. **C** PET image. All images are in sagittal view.

**Figure 2:**
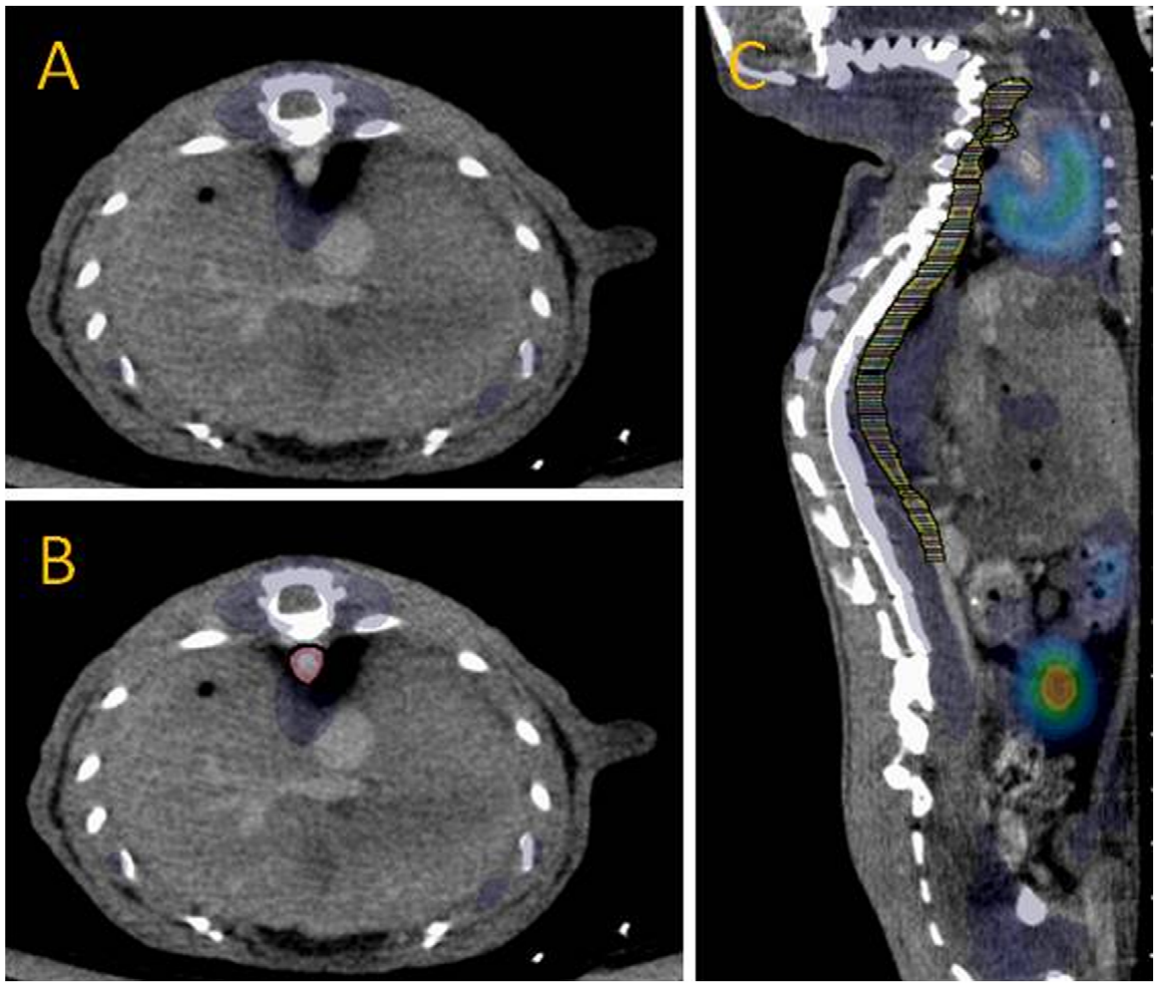
Selection of ROIs. **A** Fused PET/CT image, axial view. **B** Fused PET/CT image with ROI drawn, axial view. **C** Fused PET/CT image with the ROIs after ROI interpolation, sagittal view. Most, but not the entire aorta is visible.

^18^F-FDG uptake in the aorta was quantified for each animal using the image analysis software Inveon Research Workplace (Siemens Medical Solutions). Image fusion was achieved by doing rigid registration (feature of the Inveon Research Workplace software) and subsequently confirmed visually on basis of three distinct fiducial markers placed directly on the animal bed within the field of view but ≥5 mm from the regions of interest (ROIs). In Figure 1, a CT, a fused PET/CT, and a PET image of a mouse is shown. The 3D ROIs were drawn on the axial slices from the CT scan intending to cover the aorta, from the heart to the kidney arteries. The aorta appears white at the CT image (Figure 2a) due to the contrast agent. In order to include the vessel wall, the ROIs were on average drawn with a radial increase of 0.13 mm (Figure 2b). About 70 slices (every fifth slice) per mouse were drawn to cover the aorta followed by ROI interpolation (Figure 2C). During subsequent analysis, standardized uptake values (SUVs) were calculated. The mean value of the radioactivity in the ROIs was used i.e. SUV_mean_.

### Gamma Counting

The tubes with the aortas were placed in the 2480 Automatic Gamma Counter, Wizard2TM3’’ (Perkin Elmer, USA). The samples were counted for 120 seconds using a designated ^18^F-protocol. The counting efficiency of the gamma counter was tested to be 0.54. During subsequent analysis, SUVs were calculated.

### RNA Extraction and Reverse Transcription

Total RNA was isolated with TRI Reagent® in accordance with the protocol of the manufacturer (Molecular Research Center Inc., USA). The quality of the isolated RNA was tested using the Agilent 2100 Bioanalyzer in conjunction with the Agilent RNA 6000 Nano Kits (Agilent Technologies Denmark A/S, Denmark). A RNA integrity number (RIN)-value above 5 was accepted [14]. The quantity of RNA was measured using the NanoDrop 1000 (Thermo Fischer Scientific, USA).

Total RNA (0.3 µg) was reversed transcribed (RT) using the AffinityScript™ QPCR cDNA Synthesis Kit (Stratagene, USA, cat.no. 600559) according to the protocol of the manufacturer. The RT was performed using the Eppendorf Mastercycler Gradient (Eppendorf AG, Germany) with the following protocol: incubation at 25°C for 5 minutes (primer annealing), 42°C for 15 minutes (cDNA synthesis) and 95°C for 5 minutes (termination of cDNA synthesis). Immediately after, the samples were cooled down and stored at −20°C.

### Identifying the Optimal Reference Genes

The optimal reference genes for the study were selected from a panel of twelve common endogenous control genes (pre-fabricated panel of primer-mixes from TATAA Biocenter, Sweden). All candidate genes were tested by quantitative real-time PCR (qPCR) in tissue representing non-atherosclerotic and atherosclerotic mice. To analyze gene expression stability, the software geNorm [15] was used. Actin beta (ACTB), beta-2 microglobulin (B2M) and glucuronidase beta (GUSB) were found to be the optimal reference genes for normalization.

### Quantitative Real-time PCR

Gene expression was quantified on the Mx3000P® and Mx3005™ real-time PCR systems (Stratagene). Primers and dual-labelled hydrolysis probes for the genes of interest and references genes were designed using Beacon Designer™ (PREMIER Biosoft, USA). The design included a BLAST search for test of sequence homology, a test for secondary structures and optimization of multiplex setup. The genes, accession number, primers, probes and amplicon lengths are listed in Table 2. All primers and probes were purchased from Sigma-Aldrich (Sigma-Aldrich Danmark A/S, Denmark). For each gene, the optimal primer and probe concentration was established. All samples were run in duplicates for genes of interest and reference genes using 1 µl of cDNA.

**Table 2:** Primers and probes.

Name	Gene ID	Forward primer	Reverse primer	Amplicon length	Probe
***Genes of interest***					
CD68	NM_09853	GTGTGTCTGATCTTGCTA	GTAGGTGTCATCGTGAAG	104	ACCGCTTATAGCCCAAGGAACA
CXCL-1	NM_008176	GCCTCTAACCAGTTCC	AGCTCATTGGCGATAG	145	ACTCCAGACTCCAGCCACAC
HIF-1α	NM_010431	TGCAGTATGAATGGAGTAA	CTGCTAATGGGAACAGATTA	101	CAGGAGCCTGAGCCCTCAAA
HIF-2 α	NM_10137	GGAACTTGAAGGGTTATTG	CTCAGAGTGTCTTTAGTAGA	107	CTTAACGCTGAGGCAACAACACA
LOX-1	NM_138648	GCTTCTTCCACTTGGTAC	GCATCAACAAATACACAGATAA	144	TGTTCATACATCTCCACCACAGTGTT
MCP-1	NM_011333	CCGTAAATCTGAAGCTAATG	AGTCCGAGTCACACTA	101	TCCACAACCACCTCAAGCACT
OPN	NM_009263	CTGTGTCCTCTGAAGAA	CTCTGCATGGTCTCC	122	TCGTCATCATCATCGTCATCATCGT
TF	NM_010171	CACGGGAAAGAAAACAAA	CTGGAGAAAATCATAGCTTG	102	CTTACTCCTTCTTCCACATCAATCG
VCAM-1	NM_011693	GACAGGAGACATGGTATTAAAG	GCCAACTTCAGTCTTAGA	106	CTCGTACACCATCCGCCAGG
VEGF	NM_001025250	GTGTGTGTATGAAATCTGTG	GAGCTGAGTGTTAGCAAA	100	ATCTTCTCAGGACAAGCTAGTGAC
***Reference genes***					
ACTB	NM_007393	GTTGGTTGGAGCAAACATC	CATGGATACTTGGAATGACTA	119	CCCAAAGTTCTACAAATGTGGCTGA
B2M	NM_009735	TACGCCTGCAGAGTTAAG	CTGGATTTGTAATTAAGCAGG	124	CGAGCCCAAGACCGTCTACT
GUSB	NM_010368	CTTGGTATCATGACTATGGG	ACTCGCTCTGGATAATCG	105	ACTCGCTCTGGATAATCG

The Brilliant Multiplex QPCR Master Mix (Stratagene, cat.no. 600553) was used. The thermal profile employed was 10 minutes of denaturation at 95°C followed by 40 cycles with denaturation for 15 s at 95°C and annealing/elongation at 60°C for 1 minute.

The quantification and normalization of results were based on the computation of target quantification cycle (Cq) values and reference gene Cq values in the qbase^PLUS^ software (Biogazelle NV, Belgium). The method of qbase^PLUS^ is a modification of the classic delta-delta-Ct method [16]. Instead of just normalizing to a single reference gene, multiple reference genes are taken into account and correction for gene specific amplification efficiencies can be done. The three reference genes, ACTB, B2M and GUSB, were included in the analysis. The reference target stability was 0.63 (M-value) and 0.27 (CV-value). This is a little higher than what is expected from homogeneous sample panels, however, as these samples include various cell types they are classified as heterogeneous and higher M- and C-values are expected. One default amplification efficiency (100%) for all targets was used, as the amplification efficiency varied little over the multiple runs. The data were reported as calibrated normalized relative quantities (CNRQs). As all the samples could not be included in a single run, inter-run calibration was done to correct for run-to-run differences. This was based on three inter-run calibrators included in all runs.

### Plasma Measurements

Cholesterol level was measured in duplicate using the kit Cholesterol Chod-Pap (Roche Diagnostics GmbH, Germany) in accordance with the protocol of the manufacturer. Calibrator (C.f.a.s from Roche Diagnostics GmbH) and controls (Wako Control Serum I and II from Wako Chemicals GmbH, Germany) were included in the analysis. The intra- and interassay coefficients of variation were 3.6% and 6.0%, respectively.

### Statistical Analysis

All statistical analyses were performed using SPSS 20.0 (IBM Corp., USA). Graphs have been constructed using GraphPad Prism version 5.0c for Mac OS X (GraphPad Software Inc., USA). All results were log-transformed to obtain Gaussian distribution as confirmed by one-sample Kolmogorov-Smirnov test. Comparisons between the different groups were performed by Student’s t-test for independent samples and Bonferroni correction of *p*-values was applied by multiplying the acquired *p*-values. Univariate linear regression was performed between the molecular markers (gene expression) and SUV_mean_-values and *p*-values were Bonferroni corrected. Markers with significant correlation (R) were subsequently included in a multivariate linear regression model with stepwise backward elimination of the least significant marker. Data are reported as mean±SEM (standard error of mean) unless otherwise indicated and *p*<0.05 was considered statistically significant.

## Results

### Uptake of ^18^F-FDG in the Vessel Wall

**Figure 3:**
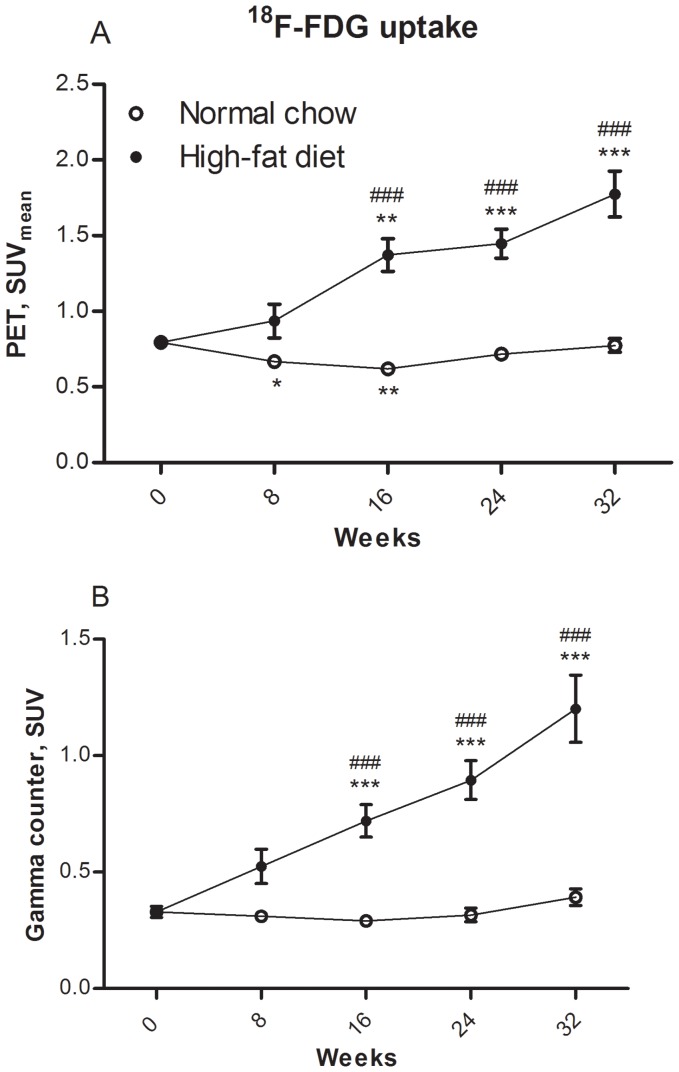
^18^F-FDG uptake assessed by SUV. ^18^F-FDG uptake assessed by SUV expressed as mean±SEM of N = 7–12. *p<0.05, **p<0.01, and ***p<0.001 are groups vs. 0 weeks group. ^##^p<0.01 and ^###^p<0.001 are high-fat diet groups vs. normal chow groups at the same age. All *p*-values were Bonferroni corrected. **A** ^18^F-FDG uptake measured by PET. **B** ^18^F-FDG uptake measured by gamma counting.

The uptake of ^18^F-FDG measured using PET and gamma counting, respectively, is shown in Figures 3a and 3b.

The uptake of ^18^F-FDG measured using PET (Figure 3a) was not significantly different in the groups receiving normal chow for 24 and 32 weeks compared to the 0 weeks group. However, the 8 and 16 weeks groups showed a small decrease in the uptake compared to the 0 weeks group with a fold change of 0.84 (p = 0.013) and 0.78 (p = 0.0012), respectively.

The high-fat Western diet had a marked effect upon the uptake of ^18^F-FDG measured by PET and from 16 weeks, SUV_mean_ was significantly higher compared to 0 weeks group. The fold change was 1.73 after 16 weeks (p = 0.0011), 1.82 after 24 weeks (p<0.001) and 2.23 after 32 weeks (p<0.001) compared to 0 weeks.

When comparing mice on high-fat Western diet with mice on normal chow of the same age, a significant higher ^18^F-FDG uptake measured by PET was seen after 16 weeks of dieting compared to non-dieting (2.21 fold; p<0.001). The ^18^F-FDG uptake was 2.02 fold higher at 24 weeks on diet compared to non-diet (p<0.001). At 32 weeks, a 2.29 fold higher level was seen (p<0.001).

The ^18^F-FDG uptake measured by gamma counting showed the same pattern (Figure 3b) as measured by PET, except, the uptake did not differ significantly in the chow-fed groups compared to the 0 weeks group. The high-fat Western diet had a marked effect upon the ^18^F-FDG uptake measured by gamma counting from 16 weeks. The fold changes were 2.19, 2.72, and 3.65 (p<0.001) for 16, 24, and 32 weeks, respectively, compared to the 0 weeks group.

The comparison of ^18^F-FDG uptake measured by gamma counting of mice on high-fat Western diet to the mice of the same age on chow also showed a significant increase from 16 weeks. The ^18^F-FDG uptake was 2.47 fold higher at 16 weeks, 2.83 fold at 24 weeks, and at 32 weeks the increase was 3.06 fold (p<0.001).

### Correlation of PET and Gamma Counting

**Figure 4:**
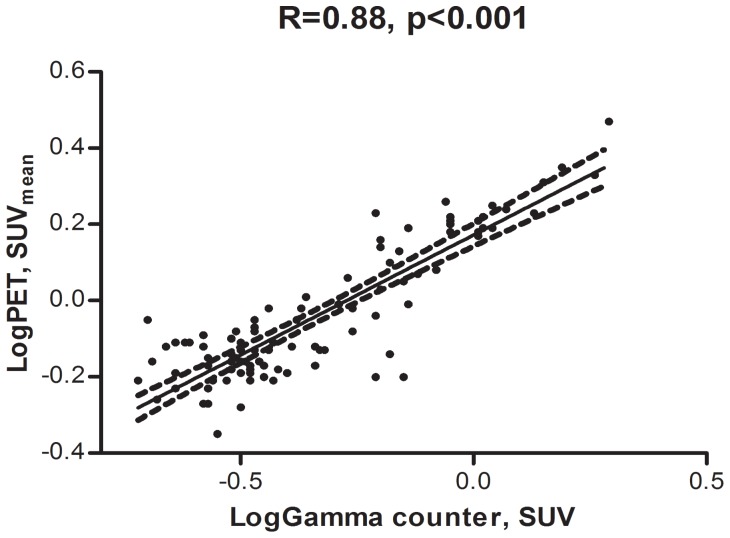
Correlation plot of PET and gamma counter data. Correlation plot of SUV values from PET and gamma counter. The 95% confidence interval is indicated by the broken lines.

A correlation plot of PET and gamma counter data is shown in Figure 4.

A strong correlation between the two methods was seen with a R-value of 0.88 (p<0.001).

### Univariate Linear Regression Analysis of Gene Expression of the Molecular Markers Relative to ^18^F-FDG SUV_mean_

In Table 3 the R-values and their *p*-values are listed for all gene expression markers.

**Table 3:** Univariate linear regression analyses of gene expression relative to ^18^F-FDG SUV_mean_.

	R	*p*-value
***Monocyte/macrophage recruitment***		
CXCL-1	0.30	0.03
MCP-1	0.46	<0.001
VCAM-1	0.61	<0.001
***Macrophages/inflammation***		
CD68	0.70	<0.001
OPN	0.60	<0.001
***Scavenger receptors***		
LOX-1	0.53	<0.001
***Hypoxia***		
HIF-1α	−0.46	<0.001
HIF-2 α	−0.59	<0.001
VEGF	−0.53	<0.001
***Thrombogenecity***		
TF	−0.71	<0.001

All *p*-values were Bonferroni corrected.

The gene expression of all markers of monocyte/macrophage recruitment exhibited significant correlations with ^18^F-FDG uptake. All correlations were positive. VCAM-1 had the highest correlation (R = 0.61, *p*<0.001). The same was true for the next group, markers of macrophages/inflammation. Both CD68 and OPN were positively correlated with SUV_mean_. CD68 exhibited the strongest correlation (R = 0.70, *p*<0.001). LOX-1, the scavenger receptor, was also positively and significantly correlated to SUV_mean_ (R = 0.53, *p*<0.001). The gene expression of all markers of hypoxia exhibited significant negative correlations with ^18^F-FDG uptake. Also TF was negatively correlated with SUV_mean_ and exhibited the strongest correlation (R = -0.71, *p*<0.001).

### Multivariate Linear Regression Analysis of Gene Expression of the Molecular Markers Relative to ^18^F-FDG SUV_mean_

**Figure 5:**
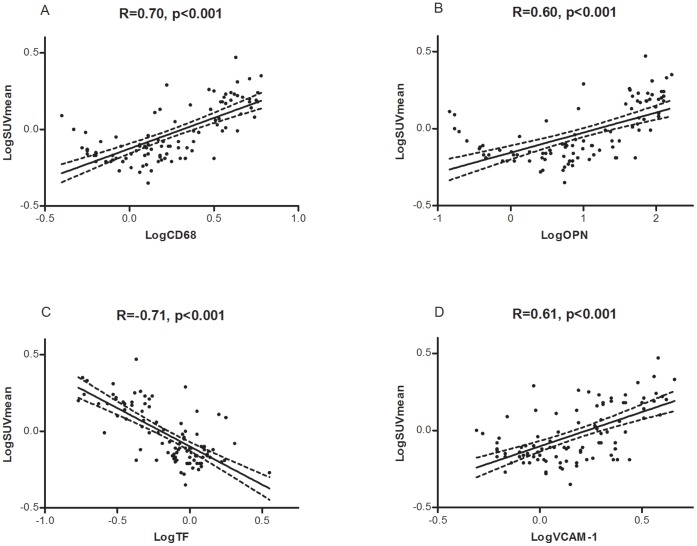
Univariate linear regression analysis of gene expression relative to SUV_mean_. Univariate linear regression analysis of gene expression relative to SUV_mean_ (N = 98). **A** CD68 relative to SUV_mean_. **B** OPN relative to SUV_mean_. **C** TF relative to SUV_mean_. **D** VCAM-1 relative to SUV_mean_. The 95% confidence interval is indicated by the broken lines. All *p*-values were Bonferroni corrected.

As all molecular markers were significantly correlated with SUV_mean_, they were all included in a multivariate analysis. After elimination, CD68 (β = 0.60, *p*<0.001), OPN (β = −0.12, *p* = 0.005), TF (β = −0.37, *p*<0.001) and VCAM-1 (β = −0.23, *p* = 0.06) remained in the final model with an R^2^ of 0.60 (p<0.001). The univariate analyses of the 4 genes left in the final model are shown in Figure 5.

## Discussion

Our study showed increased uptake of ^18^F-FDG in apoE^−/−^ mice on high-fat diet compared to mice on normal chow and the uptake increased with duration of diet. This supports recent findings, that ^18^F-FDG can be used to follow the progression of atherosclerosis in mice [9]. To further validate the image-based method, we also performed *ex vivo* gamma counting of all vessels that were PET-imaged. *Ex vivo* counting is devoid of any partial volume and spillover effects and may be considered a reference for tracer uptake. Indeed the two patterns of FDG uptake was in essence identical proving that when using our protocol for animal handling and imaging, spillover is of no concern. Accordingly, a strong correlation between the two methods was found. Secondly, we identified 4 genes involved in atherogenesis that together explained 60% of the ^18^F-FDG uptake.

^18^F-FDG is a glucose analogue taken up by metabolic active cells [17] and evidence suggests that uptake in atherosclerotic lesions correlates with vascular inflammation. Macrophages are thought to be the dominant cell type responsible for the uptake, although it has not been established conclusively [6], [18].

To examine the molecular processes contributing to the uptake of ^18^F-FDG in the aortas of apoE^−/−^ mice, we investigated the gene expression of 10 molecular markers representing different molecular processes important in the atherogenesis. Surprisingly, we found that all of these markers had a significant correlation with the uptake of ^18^F-FDG assessed as SUV_mean_.

### Monocyte/macrophage Recruitment

CXCL-1 and MCP-1 are chemokines important for the recruitment of immune cells to sites of tissue injury and infection. Both chemokines are produced by a variety of cells and have been demonstrated in human atherosclerosis [19], [20]. We found a rather low correlation of SUV_mean_ with CXCL-1 and MCP-1. Studies in knockout mice suggested that CXCL-1 and its receptor is important after early lesions have been established [21] which implies a delayed expression relative to the onset of atherosclerosis whereas ^18^F-FDG uptake is increased from early in the atherogenesis (Fig. 3). Therefore, it seems reasonable that the expression of CXCL-1 does not correlate perfectly with SUV_mean_. MCP-1 on the other hand, has been shown to be crucial for the initiation of atherosclerosis [22]. The relative low correlation with SUV_mean_ may reflect that MCP-1 decreases with the progression of disease. A study of MCP-1 gene expression in mice showed a peak after only 10 weeks of high-fat dieting [23] further supporting this. VCAM-1 is an inducible endothelial cell surface molecule playing a role in mononuclear cell attachment, rolling, and transendothelial migration and is found to be important in both human and murine atherosclerosis [24], [25]. VCAM-1 had a high correlation with SUV_mean_ (Fig. 5d). This finding suggests that the expression of VCAM-1 is critical for metabolic activity of the tissue throughout atherogenesis. VCAM-1 also remained in the final model from the multivariate linear regression analysis, further emphasizing its independent importance for ^18^F-FDG uptake. A mouse study showed increasing presence of VCAM-1 with high-fat diet and age [26]; which is in line with our findings.

### Macrophages/inflammation

As expected, markers of macrophage/inflammation showed a very strong correlation with SUV_mean_ (Fig. 5a and 5b). CD68 is a scavenger receptor expressed by macrophages, Langerhans cells, dendritic cells, and osteoclasts. It is widely used as marker for macrophage activity as it is significantly up-regulated in activated macrophages [27], [28]. The strong correlation between CD68 and SUV_mean_ was expected, as numerous studies, both clinical and pre-clinical, have found a high degree of correlation between macrophages and uptake of ^18^F-FDG [1], [7], [8], [29]. OPN is a secreted protein expressed by several cell types during inflammation. It is highly expressed at sites with atherosclerotic plaques and present in human atherosclerosis [30]. The expression of OPN has to our knowledge not previously been correlated with ^18^F-FDG uptake. The high degree of correlation we found is supported by studies of knockout mice suggesting OPN as an important player in inflammation and atherogenesis [31], [32]. Both CD68 and OPN had independent predictive value for ^18^F-FDG uptake in the multivariate linear regression analysis.

### Scavenger Receptors

LOX-1 is expressed by endothelial cells, macrophages, smooth muscle cells, and platelets and is present in human atherosclerosis [27], [33]. We found that LOX-1 had a relatively strong positive correlation with SUV_mean_. Our finding is supported by previous studies of knockout mice. Here, a reduction of macrophages and a reduced expression of OPN when the mice were deficient of LOX-1 were found [34], [35].

### Hypoxia

Hypoxia has been demonstrated in advanced human atherosclerosis [36]. HIF-1α and HIF-2α are often used as markers of hypoxia and although the genes are similar, they are not identical, as the list of genes they regulate is only overlapping, not identical. HIF-1α is expressed ubiquitously, whereas HIF-2α is only expressed in certain tissues [37], [38]. VEGF is regulated by both HIF-1α and HIF-2α. Whereas the expression of VEGF is low in normal vessel wall, it is up-regulated by hypoxia, inflammatory mediators, and certain growth factors. In atherosclerosis, VEGF is thought to contribute to inflammation, to intimal thickening, and to intra-plaque angiogenesis [39]. We found that all three markers of hypoxia correlated negatively with SUV_mean_. This is in contrast to a human study from 2008 [36], where the researchers demonstrated up-regulated expression of the same markers comparing stable plaques with early lesions and comparing thrombus-containing plaques with stable plaques, that is to say increasing expression over the development of atherosclerosis. The significance of the results was limited and as the authors only investigated a limited number of lesions (5 of each category), it could be argued that the results may not be representative. Also, we did not study distinct plaques but rather diffuse atherosclerotic disease. Another explanation of the discrepancy to the findings in our study could arise from the smaller lesions in mice compared to humans. It may be that hypoxia is limited in murine atherosclerosis; therefore, inferring a dissimilar gene expression of molecular markers of hypoxia compared to humans is logical [40].

### Thrombogenicity

TF is expressed both by cells in the vessel wall and by platelets. TF is not only an important player in thrombogenicity, but also functions as a membrane receptor which activation leads to cell proliferation, angiogenesis, and inflammation [41]. We found a strong negative correlation with ^18^F-FDG SUV_mean_ and that TF had independent predictive value for SUV_mean_ in the multivariate linear regression analysis. The negative correlation with SUV_mean_ implies that TF decreases with increasing uptake of ^18^F-FDG indicating that TF expression decreases with the progression of atherosclerosis. This is in discrepancy with a human study from 1997 [42] where an increase in TF activity was seen during the progression of disease. However, as we were investigating mRNA expression instead of TF activity, this may at least in part explain the different results. Murine studies of TF in atherosclerosis have been sparse and not unanimous, suggesting all from no importance to influencing the degree of atherosclerosis [43]–[45]. Taken together, the role of TF in murine atherosclerosis requires further studies and studies of the mRNA levels in human atherosclerotic lesions will also be of importance.

The present study has some limitations for consideration. The use of PET as a technique for measuring uptake of ^18^F-FDG in small areas such as the aorta of mice involves certain challenges, such as resolution and partial volume effects. As all the animals have been treated similarly, the problems are presumed to be alike and the relative comparisons we have made are therefore expected to be largely unaffected. This is also supported by the high correlation found between *ex vivo* gamma counting of the aortas and PET scans. We measured the expression of mRNA in preference to protein levels and it cannot be assumed that these levels are totally consistent as e.g. post-transcriptional regulation may be prominent [46]. The mRNA measurements were preferred as these are easily quantifiable compared to measurements using immunohistochemistry.

### Conclusion

In this study, we have demonstrated that ^18^F-FDG can be used to follow the progression of atherosclerosis in apoE^−/−^ mice. The gene expression of ten molecular markers representing different molecular processes important for atherosclerosis was shown to correlate significantly with the uptake. A multivariate analysis showed CD68, OPN, TF, and VCAM-1 to be the most important contributors and a statistical model with these parameters explained 60% of the ^18^F-FDG uptake.
